# Protective effect of polydatin on radiation-induced injury of intestinal epithelial and endothelial cells

**DOI:** 10.1042/BSR20180868

**Published:** 2018-11-14

**Authors:** Li Li, Ke Zhang, Ji Zhang, Ya-Nan Zeng, Feng Lai, Gen Li, Na Ma, Ming-Jiang Hu, Feng-Mei Cui, Qiu Chen

**Affiliations:** 1 Department of Oncology, the First Affiliated Hospital of Suzhou University, Suzhou 215004, China; 2 State Key Laboratory of Radiation Medicine and Protection, School of Radiation Medicine and Protection, Soochow University, Suzhou 215123, China; 3 Collaborative Innovation Center of Radiation Medicine of Jiangsu Higher Education Institutions, Suzhou 215123, China; 4 Department of Ophthalmology, the Second Affiliated Hospital of Suzhou University, Suzhou 215004, China; 5 Department of Occupational Health, Wuxi Center for Disease Control and Prevention, Wuxi 214023, China

**Keywords:** irradiation, intestinal epithelial cells, intestinal endothelial cells, polydatin (PD)

## Abstract

This study aimed to examine the radioprotective effect of polydatin (PD) on crypt and endothelial cells of the small intestines of C57BL/6 mice that received abdominal irradiation (IR). Mice were treated with 6 MV X-ray (20 Gy) abdominal IR at a dose rate of 200 cGy/min. Thirty minutes before or after IR, mice were intraperitoneally injected with PD. The rate of survival of the mice at 30 days after IR was determined. The duodenum (upper small intestine), jejunum (middle small intestine), and ileum (lower small intestine) were collected and subjected to hematoxylin and eosin staining. Tissue sample sections were analyzed through light microscopy, and the lengths of at least 20 intestinal villi were measured in each group; the average number of crypts was obtained from 10 intestinal samples in each group. Microvessel density was assessed using CD31-positive (brown) vascular endothelial cells/cell clusters. FHs74Int cell proliferation was measured using the CCK-8 assay. PD administration (25 mg/kg) before IR was the most effective in prolonging the survival of C57BL/6 mice. PD reduced radiation-induced injury of intestinal villi, prevented loss of crypts, increased intestinal crypt growth, protected against IR-induced intestinal injury, and enhanced the proliferative potential and reduced the apoptosis of FHs74Int cells after IR. Moreover, PD increased small intestinal MVD and reduced the apoptosis of intestinal microvascular endothelial cells in mice after IR. Therefore, PD was found to be able to protect the two types of cells from radiation damage and to thus alleviate radiation-induced injury of small intestine.

## Introduction

Gastrointestinal syndrome can be induced in cancer patients undergoing abdominal radiotherapy and in people accidentally exposed to abdominal irradiation (IR) [1]. Currently, symptomatic treatment is the only course of therapy due to the lack of effective drug-based therapies. There is, therefore, an urgent need to develop novel drugs that are effective in treating radiation-induced gastrointestinal syndrome [1].

To date, various radioprotective compounds have been identified, such as sulfur compounds, biogenic amines, estrogens, natural compounds (e.g., polysaccharides, coumarins, and alkaloids), and cytokines [2–7]. Unfortunately, amifostine is the only radioprotective agent currently approved by the U.S. Food and Drug Administration (FDA) for the prevention of radiation-induced severe toxic reactions. However, the clinical application of amifostine is limited owing to its hematotoxicity and gastrointestinal toxicity [8]. Recently, low-cost, non-toxic compounds derived from plants have received increased interest. For example, polydatin (PD) is claimed to exhibit a wide spectrum of biomedical properties, including antiplatelet aggregation, antioxidant activity, cardioprotective effects, anti-inflammatory properties, and antitumor and immunomodulatory functions [9–11]. However, the possible radioprotective effect of PD on radiation-induced intestinal injury has not yet been investigated.

Small intestinal mucosa undergoes rapid renewal and is radiosensitive. Epithelial cells in this mucosa, especially crypt stem cells, are highly sensitive to the cell killing effects of ionizing radiation, which can lead to an insufficient supply of intestinal epithelial cells, villus exfoliation, shrinkage or disappearance of crypts, and mucosal damage [12,13]. Despite considerable research, the molecular mechanisms underlying radiation-induced gastrointestinal toxicity and gastrointestinal syndrome-induced cell death remain controversial [14]. Some studies have suggested that radiation-induced damage to capillary endothelial cells of the intestinal mucosa causes gastrointestinal syndrome [15]. Other studies have reported that endothelial cell damage is not sufficient to cause crypt cell death, and that endothelial cell apoptosis plays a role in gastrointestinal syndrome [16,17]. The general consensus is that both epithelial and endothelial cells in the crypts are involved in radiation-induced intestinal injury: the epithelial cells are the most important target of radiation damage, which may subsequently induce apoptosis in endothelial cells [18,19].

In the present study, we examined the radioprotective effect of PD on crypt cells and endothelial cells of the small intestines of C57BL/6 mice that had received abdominal IR. Our observations were evaluated to provide an experimental basis for the clinical use of PD against radiation-induced intestinal injury and gastrointestinal syndrome.

## Materials and methods

### Reagents and instruments

PD was obtained from the Meilun Biological Company (China). Xylene, ethanol, and ethylenediaminetetraacetic acid (EDTA) were purchased from the Sinopharm Chemical Reagent Co., Ltd. Anti-Ki-67 antibody was purchased from the BD Company, cleaved caspase-3 antibody was purchased from the Cell Signaling Company, and CD31 antibody was purchased from Abcam. The FHs74Int cells were obtained from the American Type Culture Collection (ATCC). The CCK-8 kit was purchased from the Biotool Company (USA). The multi-functional microplate reader was obtained from Molecular Devices (USA). The microscope was purchased from Olympus (Japan).

### Animals

Six-week-old male C57BL/6 mice weighing 20–25 g were purchased from Shanghai SLAC Laboratory Animal Co., Ltd (China) and housed at the Animal Center of Suzhou University under specific-pathogen-free conditions. All animal experiments were approved and conducted in accordance with the guidelines of the ethics committee of Soochow University (number ECSU-201800053).

### Radiation and survival analysis

Thirty minutes before or after IR, mice were intraperitoneally injected with PD at different doses (25 mg/kg, 50 mg/kg, and 100 mg/kg). Mice were randomly divided into six groups: IR; 25 mg/kg PD+IR; 50 mg/kg PD+IR; 100 mg/kg PD+IR; IR + 25 mg/kg PD; and IR + 50 mg/kg PD. Mice were treated with 6 MV X-rays (20 Gy) abdominal IR at a dose rate of 200 cGy/min from a linear accelerator in Suzhou Jiulong Hospital. The rate of survival of the mice at 30 days after IR was determined.

### Sample collection

Mice were sacrificed 1 week after IR. The duodenum (upper small intestine), jejunum (middle small intestine), and ileum (lower small intestine) were collected and fixed in 10% formalin for 12 h. The tissues were then trimmed, dehydrated, embedded in paraffin, and sectioned.

### Hematoxylin and eosin (H&E) staining

Tissue sections were baked in an oven at 70°C for 30 min, deparaffinized with three washes in xylene for 5 min each, rehydrated through an ethanol series (100%, 95%, 90%, and 85%, each for 1 min), and stained with hematein for 3 min. The sections were washed, immersed in 100% ethanol for 1 min, and counterstained with eosin for 20 s. After staining, sections were dehydrated through an ethanol series (75%, 80%, 85%, 90%, 95%, and 100%, each for 1 min), mounted on glass microscope slides, and observed using a light microscope.

### Intestinal villus measurement

Tissue sample sections were analyzed through light microscopy (200× magnification) and the lengths of at least 20 intestinal villi were measured in each group.

### Number of small intestinal crypts

Tissue sample sections were analyzed through light microscopy (200× magnification) and the average number of crypts was obtained from counts of 10 intestinal samples in each group.

### Immunohistochemistry

Tissue sections were baked in an oven at 70°C for 60 min, deparaffinized with three washes in xylene for 5 min each, rehydrated through an ethanol series (100%, 95%, 90%, and 85%, each for 5 min), washed twice in distilled water for 5 min each, immersed in Tris-EDTA buffer for antigen retrieval, washed three times in phosphate-buffered saline (PBS) with Tween 20 (PBST) for 5 min, blocked in 5% bovine serum albumin for 1 h, and incubated with CD31 (1:200) and Ki67 (1:200) antibodies overnight at 4°C. Subsequently, the sections were washed three times in PBST for 10 min each and incubated with an HRP-conjugated secondary antibody at room temperature for 30 min. The sections were washed three times in PBST for 10 min each, subjected to the diaminobenzidine (DAB) reaction, stained in hematoxylin for 40 s, dehydrated through 85%, 90%, 95%, and 100% ethanol, mounted on glass microscope slides, and observed under a light microscope.

### Microvessel density (MVD) measurement

MVD was assessed using Widmer’s counting method^19^ using CD31-positive (brown) vascular endothelial cells/cell clusters that were distinct from the background. Vessel lumen formation was not necessary for determination of MVD, and blood vessels with an obvious muscular layer were excluded. A total of 10 fields of view (400×) were randomly selected in each mouse and used to calculate MVD.

### Double immunohistochemistry

Sections of small intestine were dried at 70°C in an oven for 1 h, deparaffinized in xylene, rehydrated through an ethanol series, washed in Tris-EDTA for antigen retrieval, and blocked in 10% goat serum for 1 h. The sections were incubated with CD31 antibody (1:200) for 1 h, stained with GBI-Permanent Red, and blocked in Blocker A/B. After three washes in PBST, the sections were incubated with cleaved caspase-3 antibody (dilution, 1:800) for 1 h, stained with Emerald Chromogen reagent, and dehydrated through an ethanol series. The sections were mounted on glass microscope slides and analyzed using a light microscope.

### Cell viability assay

FHs74Int cells were cultured in Hybri-Care medium supplemented with 10% FBS and human EGF (30 ng/ml) in an incubator at 37°C and 5% CO_2_. The cells were seeded in a 96-well plate at a density of 5000 cells/well. After adherence, the cells were incubated with different doses of PD (0, 20, 40, 60, 80, 100, and 120 μM) for 24, 48, and 72 h. Then, FHs74Int cells were treated with CCK-8 (10 μl/well) for 2 h at 37°C and absorbance at 450 nm was measured to determine the cytotoxicity of PD. In a subsequent experiment, FHs74Int cells were incubated with PD for 30 min, treated with X-rays (10 Gy), and cultured for 24, 48, and 72 h. FHs74Int cell proliferation was measured using the CCK-8 assay.

### Apoptosis determination

The FHs74Int cells were seeded in 60-mm plates at a density of 5×10^5^ cells/well. The FHs74Int cells were incubated with PD (20 μM) for 30 min, treated with X-rays (10 Gy), and cultured for 48 h. The cells were trypsinized, pelleted, and re-suspended in PBS. A total of 1–5×10^5^ cells were incubated with Binding Buffer (500 μl), Annexin V-FITC (5 μl), and propidium iodide (5 μl) for 5–15 min in the dark. Within 1 h, cells were subject to flow cytometry.

### Statistical analysis

Survival analysis was performed using the Kaplan–Meier method. Expression of Ki67 in different groups was compared using the CMHx2 test. Other experimental data are presented as means ± standards deviation ($\bar{\text{x}}\pm \text{s}$) and differences between groups were analyzed using *t*-tests. *P* < 0.05 was considered statistically significant.

## Results

### PD treatment before and after irradiation prolongs survival time in C57BL/6 mice

**Figure 1:**
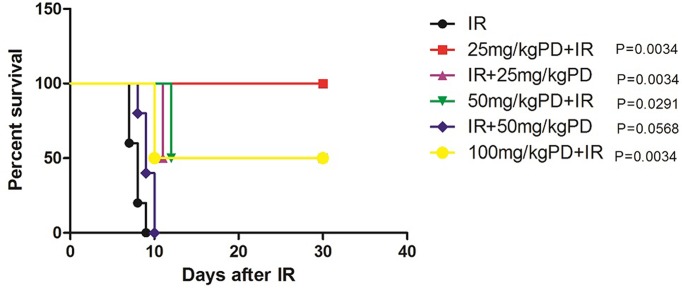
Survival of C57BL/6 mice after IR IR: mice given 20 Gy X-rays; 25 mg/kg PD+IR: mice treated with 25 mg/kg PD 30 min before IR (*P* = 0.0034 vs. IR); IR+25 mg/kg PD: mice treated 25 mg/kg PD 30 min after IR (*P* = 0.0034 vs. IR); 50 mg/kg PD+IR: mice treated with 50 mg/kg PD 30 min before IR (*P* = 0.0291 vs. IR); 100 mg/kg PD+IR: mice treated with 100 mg/kg PD 30 min before IR (*P* = 0.0034 vs. IR); IR+50 mg/kg PD: mice treated with 50 mg/kg PD 30 min after IR (*P* = 0.0568 vs. IR). Each group consisted of five mice treated with 20 Gy X-rays at a dose rate of 200 cGy/min. Survival was analyzed at 30 days after IR.

Pre-treatment with PD (25, 50, or 100 mg/kg) or post-treatment with PD (25 mg/kg) significantly prolonged the survival time of mice treated with 20 Gy X-rays (*P* < 0.05; Figure 1). However, post-treatment with 50 mg/kg PD had no effect on survival time after IR (*P* < 0.05). Overall, therefore, PD administration before IR was more effective in prolonging the survival time of C57BL/6 mice. As treatment with 25 mg/kg PD was the most effective (Figure 1), we selected this concentration for the subsequent experiments.

### PD reduces radiation-induced injury of intestinal villi and loss of crypts

**Figure 2:**
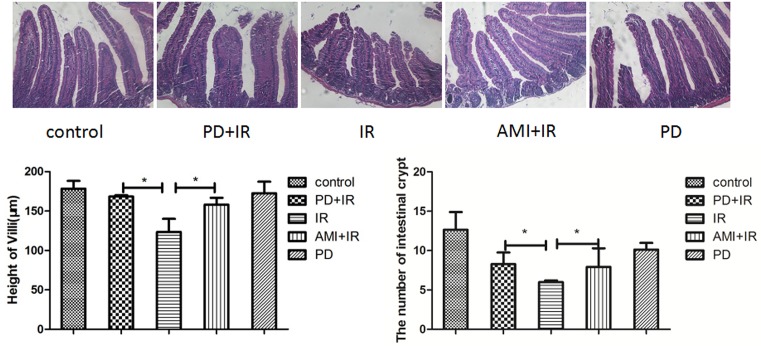
Effect of PD on villus length and crypt number in the small intestine *, *P* < 0.05. 200× magnification. Intestinal villus length was measured in at least 20 villi in each intestinal canal. The average number of intestinal crypts was calculated from counts upon observation under a light microscope (200×) of 10 intestinal segments in each group. PD dosage, 25 mg/kg. PD, polydatin; IR, X-irradiation; AMI, amifostine.

IR of mice with X-rays (10 Gy) induced apical rupture of villi, detachment of villi, and shortened villus lengths (by about 50 μm) in the small intestine (Figure 2). Treatment with PD before X-irradiation reduced the level of radiation injury in terms of villus structure and length compared with that in mice that only received the radiation treatment. Villus length increased by about 48 μm in the PD+IR group compared to that in the IR group. The number of crypts in the small intestine of IR group mice was reduced compared to that in the PD+IR group (*P* < 0.05). These findings, to a certain extent, suggest that PD can alleviate radiation-induced intestinal injury.

### PD promotes an increase in intestinal crypt numbers after X-irradiation

**Figure 3:**
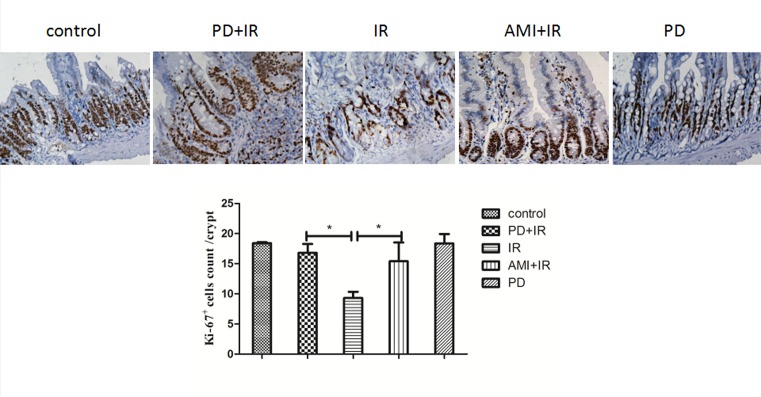
Immunohistochemical staining of small intestinal crypt with Ki67 antibody *, *P* < 0.05. 400× magnification. PD dosage, 25 mg/kg.

The average number of Ki67^+^ crypt cells in the X-irradiated group (9 ± 1) was significantly lower than in the untreated, control group (18 ± 1) (Figure 3). The number of Ki67^+^ crypt cells increased in the PD+IR group (16 ± 1) compared to that in the IR group. Treatment with amifostine (AMI) before IR increased Ki67^+^ crypt cell number (15 ± 1) compared to that in the IR group; PD treatment alone did not affect the number of Ki67^+^ crypt cells compared to the untreated control. Overall, PD was found to increase intestinal crypt growth and protect against IR-induced intestinal injury.

### PD promotes FHs74Int cell proliferation and prevents apoptosis after X-irradiation

**Figure 4:**
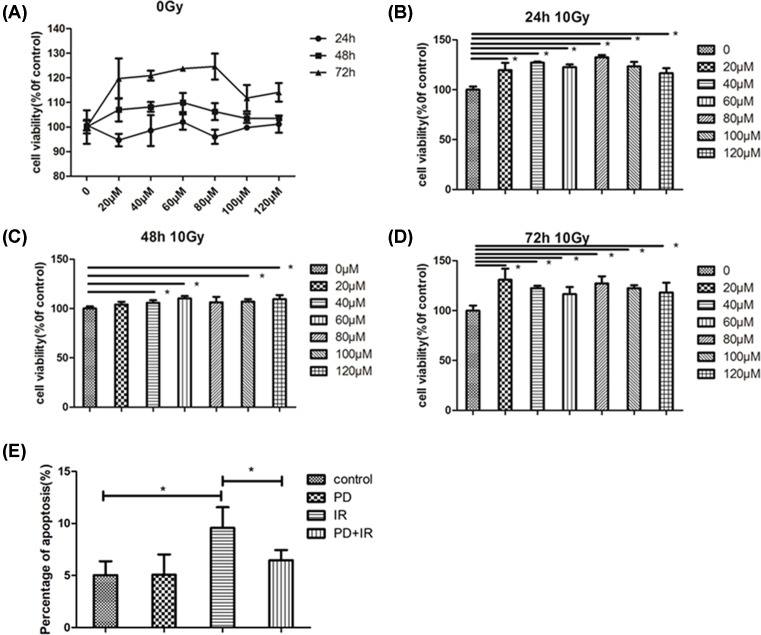
Effect of PD on FHs74Int cell proliferation and apoptosis (**A**) Cytotoxicity of PD at various concentrations to FHs74Int cells. Effect of PD treatment on FHs74Int cell survival at 24 h after IR at 10 Gy (**B**), 48 h (**C**) and 72 h (**D**). (**E**) Effect of PD on the FHs74Int cell apoptosis at 48 h after IR. *, *P* < 0.05.

Intestinal epithelial cells (FHs74Int) were cultured to investigate the effect of PD on their proliferation and apoptosis. As shown in Figure 4A, PD at the tested concentrations (20, 40, 60, 80, 100, and 120 μM) had no cytotoxicity on FHs74Int cells. Twenty-four hours after IR (10 Gy), PD at the tested doses increased the rate of survival compared to that of irradiated control cells (Figure 4B); similarly, at 48 h after IR, most PD concentrations increased the rate of cell survival (Figure 4C); at 72 h after IR, PD continued to increase the rate of cell survival (Figure 4D). Apoptosis was detected at 48 h after IR, and the rate of apoptosis is shown in Figure 4E. The results demonstrated that PD can reduce the occurrence of apoptosis in irradiated FHs74Int cells. Overall, PD at the tested doses enhanced the proliferative potential and prevented the apoptosis of FHs74Int cells after IR.

### PD increases small intestinal MVD in mice after IR

**Figure 5:**
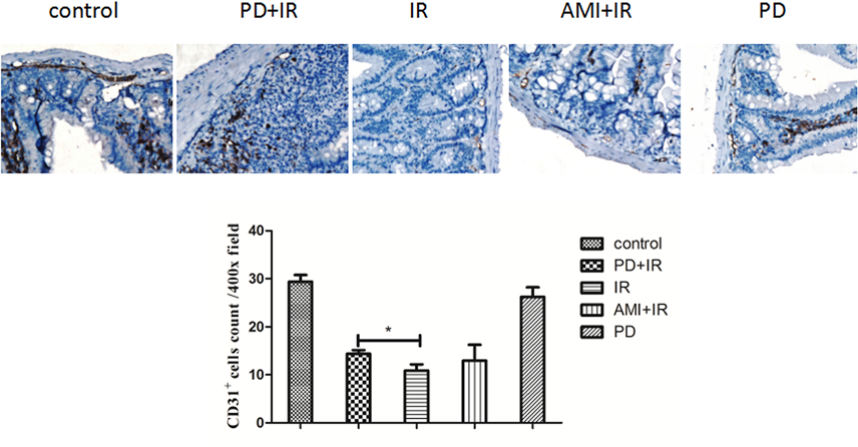
Immunohistochemical staining of small intestine with CD31 antibody *, *P* < 0.05. 400× magnification. Blood vessels were identified in vascular endothelial cells/cell clusters (brown) and were distinct from the background. A total of 10 fields of view (400×) were randomly selected in each mouse to determine MVD. PD dosage, 25 mg/kg.

As can be seen in Figure 5, MVD in each analyzed field of view was lower in the IR group mice (10 ± 1) than in the unirradiated control mice (29 ± 1). Treatment with PD increased MVD (15 ± 1; *P* < 0.05), suggesting that PD had a radioprotective effect on intestinal microvascular endothelial cells. Here, AMI showed no radioprotective effect.

### PD reduces IR-induced apoptosis of intestinal microvascular endothelial cells

**Figure 6:**
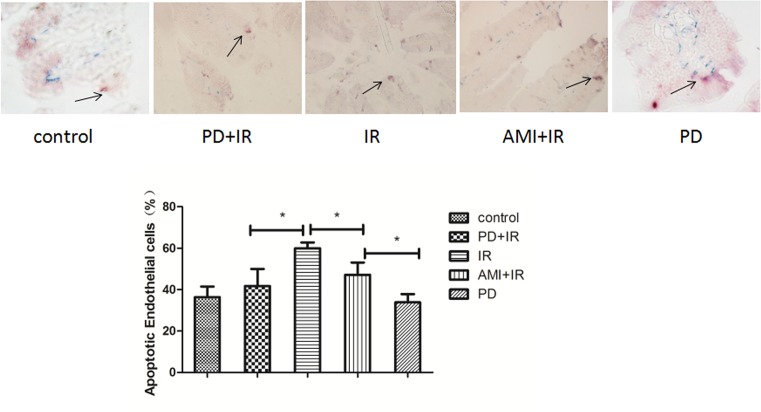
CD31/cleaved caspase-3 double immunohistochemical staining of small intestine in mice *, *P* < 0.05. 400× magnification. CD31^+^, green; cleaved caspase-3^+^, red. Purple, CD31^+^/cleaved caspase-3^+^ with higher expression of cleaved caspase-3 than CD31 (black arrow). Blue, CD31^+^/cleaved caspase-3^+^ with higher expression of CD31 than cleaved caspase-3. PD dosage, 25 mg/kg.

The rate of apoptosis in intestinal microvascular endothelial cells was lower in the PD+IR group (42.3%) than that in the IR group (58.8%) (Figure 6), suggesting that PD may have aided the functional recovery of the small intestine by reducing IR-induced apoptosis of intestinal microvascular endothelial cells.

## Discussion

PD is derived from Japanese knotweed (*Polygonum cuspidatum*); it is a resveratrol glycoside with the chemical formula 3,4,5′-trihydroxy-stilbene-3-β-d-mono-d-glucoside. The compound has been found in many other plants (12 families, 31 genera, and 72 species) including grape, peanut, hop cones, and in cocoa-containing products [20]. PD has been claimed to exhibit a wide range of potential applications in biomedicine and has low cost and toxicity [9–11]. A great number of pharmacological and pharmacokinetic investigations have demonstrated that PD shows many pharmacological effects, including cardiovascular protection, neuroprotection, immunoregulation, antioxidation, antitumor, and lung protective effects. PD has favorable therapeutic properties, indicating its potential as an effective material.

Radiation-induced gastrointestinal syndrome generally occurs 6–10 days after IR. The symptoms include nausea, vomiting, anorexia, inflammation, diarrhea, abdominal cramps and pain, hematochezia, malabsorption, and even death in the most severe cases due to associated complications such as infection, dehydration, and electrolyte imbalance [1]. The pathogenesis is believed to be associated with radiation-induced injury of the intestinal mucosa and other types of cells.

The intestinal epithelium is composed of crypts and villi; stem cells reside in the basal parts of intestinal crypts and play an important role in the homeostasis and renewal of the intestinal epithelium after injury [21–23]. Crypt stem cells are highly sensitive to ionizing radiation, and exposure to radiation can lead to an insufficient supply of intestinal epithelial cells, villus exfoliation, shrinkage or disappearance of crypts, and mucosal damage [12,13]. Paris et al. [15] reported that microvascular endothelial apoptosis is the primary lesion in radiation damage and leads to stem cell dysfunction; this study showed that microvascular endothelial cells, not epithelial cells, are the primary target of radiation-induced gastrointestinal syndrome. Additionally, they found that instead of mitosis-related cell death, apoptosis induces endothelial cell death [15]. A subsequent report suggested that early apoptosis of endothelial cells (4 h after radiation) is the primary lesion in radiation-induced damage to the gastrointestinal tract [24]. However, another study indicated that endothelial apoptosis may not be induced in the gastrointestinal tract after selective vascular IR and their findings did not support the hypothesis that vascular endothelial cell apoptosis is the cause of gastrointestinal syndrome [25]. Rather, this study concluded that endothelial injury was a consequence of radiation-induced damage of normal tissues and was not an initiating factor. These various studies showed that radiation can induce damage to both intestinal epithelial and endothelial cells. Our study demonstrates that PD can increase crypt epithelial cell proliferation after IR, increase MVD, and reduce radiation-induced endothelial cell apoptosis. Thus, PD can reduce radiation-induced damage by protecting intestinal epithelial and endothelial cells.

In summary, PD can protect many types of cells from radiation damage and thus alleviate radiation-induced injury of the small intestine. Our study suggests that PD has the potential to be developed as a novel therapy against radiation-induced intestinal injury.

## Abbreviations

DAB: diaminobenzidine
EDTA: ethylenediaminetetraacetic acid
IR: irradiation
MVD: microvessel density
PBS: phosphate-buffered saline
PD: polydatin
